# Cell–Material Interactions 2022

**DOI:** 10.3390/ijms24076057

**Published:** 2023-03-23

**Authors:** Axel T. Neffe

**Affiliations:** Institute of Active Polymers, Helmholtz-Zentrum Hereon, Kantstrasse 55, 14513 Teltow, Germany; axel.neffe@hereon.de

Cell–material interactions are the defining feature of biomaterials, and they are relevant for evaluating material residues and pollutants. Studying cell–material interactions is therefore a key step in biomaterial design, as well as in the biological evaluation of biomaterials and materials interacting with biological systems after use or as residues (see Figure 1).

Biomaterial design uses as inputs the results from the analysis of the composition, structure, and properties of tissues; the elucidation of biological and physiological processes; handling requirements and limitations; opportunities in synthesis and processing; and the specification of required functionalities. The generated biomaterial has to be biologically evaluated in vitro and, for most applications, in vivo [1]. Only materials passing both functional and toxicity tests for a certain application can be categorized as biocompatible in view of the studied application—biocompatibility is not a material property [2].

In view of the diverse types of study in the described process, scientists of many different backgrounds are involved such that biomaterial science is an inherently interdisciplinary field. This is reflected in this Special Issue on “Cell–Material Interactions 2022”, in which the full spectrum of activities in biomaterial science is represented.

First, there are two reports on cellular behavior that is affected in the presence of materials. Uzieliene et al. report on the effect of external mechanical stress on the chondrogenic responses of human bone marrow-derived stem cells (BMMSC) and chondrocytes encapsulated in chondroitin sulfate–tyramine and gelatin-based hydrogels [3]. They conclude that the encapsulation allows for the induction of chondrogenic responses and that the BMMSCs were less affected by external mechanical stress than chondrocytes, highlighting the importance of the regulation of iCa^2+^ channels from a mechanistic point of view. This lays an important basis for the selection of suitable cell-material combinations for treating articular cartilage lesions. Guiding the behavior of endothelial cells by spatially controlled adhesion (based on the peptidic YISGR sequence) and angiogenic functional motifs (vascular endothelial growth factor, VEGF) was studied by Le Bao et al. [4]. For this purpose, they developed a locally controlled attachment of molecules applied to hydrogels using electrostatic interactions. The co-presentation of both motifs resulted in pro-angiogenic materials.

Two manuscripts concentrate on the material side. Munawar and Schubert focus on the processing operations for yielding highly oriented electrospun fibers based on polylactide and polyaniline [5]. They used thermal-induced percolation to great effect, increasing the conductivity of the fibers as well as Young’s modulus, which may be of relevance, e.g., in cardiac tissue engineering. In a review by Rai et al. [6], the involvement of glycosaminoglycans (GAGs) and proteoglycans (PGs) in thoracic aortic aneurysms and dissection was discussed systematically. While there is substantial evidence for GAG and PG involvement in pathological processes, clear cause-and-effect relationships still have to be established. However, even without such a defined mechanism, both GAGs and PGs might serve as prognostic or diagnostic biomarkers.

Biological evaluations, especially concerning the toxicity, were the topic of potentially respirable polyacrylamides [7,8], as well as caffeine–cyclodextrin host–guest complexes [9]. The studies on polyacrylamides highlight that organic compounds may also cause pulmonary fibrosis with long-term effects and that polymer structural features such as molar mass or crosslinking might contribute to the overall toxic effect. Caffeine–cyclodextrin complexes led to a more than additively increased toxicity compared to the single components, which may however also be related to enhanced bioactivity. Such an effect, especially when combined with other effects often observed in cyclodextrin complexes such as changed taste, suggests interesting applications in the food industry.

Finally, two reviews highlight applications, also taking into account cell–material interactions. Le et al. report the current state of quantum dots and their interaction with biological systems [10], including mammalian, fungal, and plant cells. In this manner, not only the application but also the environmental impact of quantum dots are discussed. Ji et al. review the detection of single cells and biomolecules by graphene-based optical sensors [11]. In particular, synthesis, biofunctionalization, and applications are highlighted.

## Figures and Tables

**Figure 1:**
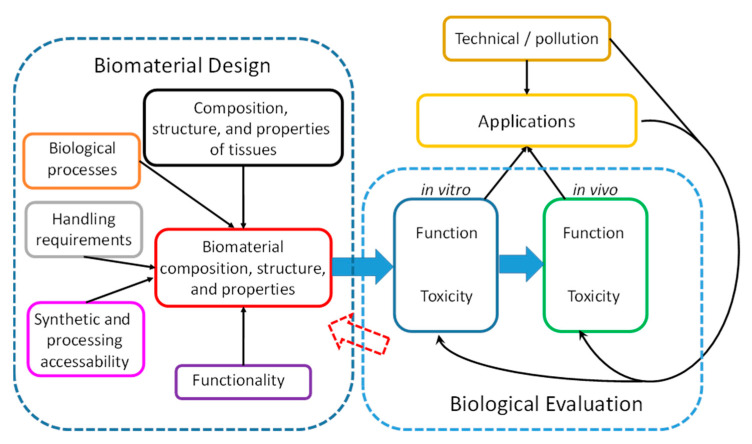
The different facets of biomaterial design and biological evaluation required for realizing biomaterial applications. Biological evaluation comprising the study of cell–material interactions provides additional important feedback for biomaterial design and is a key step in the evaluation of materials that come into contact with biological organisms after use or as pollutants.
